# Characteristics of Simulation-Based Point-of-Care Ultrasound Education: A Systematic Review of MedEdPORTAL Curricula

**DOI:** 10.7759/cureus.22249

**Published:** 2022-02-15

**Authors:** Jaskaran Singh, Lukas H Matern, Edward A Bittner, Marvin G Chang

**Affiliations:** 1 Anesthesiology and Critical Care, MedStar Georgetown University Hospital, Washington, DC, USA; 2 Anesthesia, Critical Care, and Pain Medicine, Massachusetts General Hospital, Harvard Medical School, Boston, USA

**Keywords:** critical care medicine, medical education, curricular development, simulation, point-of-care ultrasound (pocus)

## Abstract

Point-of-care ultrasonography (POCUS) is increasingly recognized as a safe, efficacious, and cost-effective diagnostic and procedural tool used by many medical disciplines. Although standardized POCUS curricula are lacking, simulation represents an effective modality to teach the fundamentals of POCUS in medical education. We sought to characterize POCUS simulation cases available within MedEdPORTAL, the primary repository of learning resources for health professions, to highlight areas for future curricular development and study.

This systematic review was performed based on a comprehensive search of MedEdPORTAL. Identified simulations were categorized and contrasted with respect to their target audiences, settings, pathologies, required materials and equipment, and POCUS techniques tested.

A total of eight curricula were identified. The majority (6/8) were targeted at trainees in acute care specialties. Pathologies included in most simulations involved cardiac or pericardial disease, although obstetric and medical diseases were also tested in isolated cases. While half (4/8) of the identified simulation curricula incorporated diagnostic POCUS interpretation, only a few (2/8) allowed for high-fidelity ultrasound simulation. While self-reported learner satisfaction appeared to be generally high, most (7/8) identified curricula did not include objective assessments of learning outcomes.

A small number of simulation-based POCUS curricula have been published within MedEdPORTAL. The widespread use of simulation for POCUS may be limited by the financial costs of high-fidelity training equipment. While simulation provides a highly promising solution to the need for greater instruction in POCUS, there is a need for comprehensive, standardized, and cost-effective curricula that can be adapted to varied educational environments.

## Introduction and background

Point-of-care ultrasound (POCUS) has assumed an increasingly prominent role in the delivery of medical care with the introduction of more affordable, portable, and versatile ultrasound devices. New diagnostic applications for ultrasound technologies, which are being developed and taught across a range of specialties, can offer immediate answers to diagnostic questions while avoiding harmful radiation exposure and reducing healthcare costs across clinical contexts and practice settings [1].

While demand for and applications of POCUS have grown, ultrasound education has not been consistently provided or standardized across medical training programs. This lack of a unified curriculum presents a significant barrier to the widespread adoption of ultrasound as a diagnostic modality, as it has been repeatedly demonstrated that operator experience is the primary limiting factor in the diagnostic and procedural utility of the technology [2]. There is an opportunity to expand clinical ultrasound education through simulation-based training, which already constitutes an important part of graduate medical education. In fact, the Accreditation Council for Graduate Medical Education (ACGME) requires both anesthesiology residents and critical care medicine fellows from various subspecialty backgrounds to participate in at least one simulated intraoperative clinical experience per year of training, while other residency programs including general surgery and emergency medicine have widely incorporated simulation into their training programs [3]. Simulation lends itself well to acute care training, as it provides opportunities to develop the reflexes and decision-making necessary to handle critical scenarios that may not otherwise be encountered during a formalized training period.

Given the demonstrated versatility and effectiveness of simulation education in developing clinical problem-solving and technical skills, the availability of standardized simulation exercises for POCUS training holds great promise. Furthermore, the literature suggests that, as the use of ultrasound across various fields expands, enthusiasm for ultrasound education among medical educators and learners continues to grow [4]. Research also indicates that high-fidelity ultrasound simulators may aid learners in rapidly and effectively achieving competency in POCUS techniques [5], although a recent systematic review on the topic of simulation-based ultrasound education suggests that the overall quality of evidence in the domain remains limited [6].

Here, we review the available ultrasound simulation cases and associated learning materials contained in the MedEdPORTAL® database, a peer-reviewed repository of instructional materials and medical education literature maintained by the Association of American Medical Colleges (AAMC). MedEdPORTAL® was selected as the ideal database for this search because of its reputation as a one-of-a-kind repository for free, open-access, and peer-reviewed curricula designed by and for medical educators [7]. The educational interventions reported in this database also uniquely provide comprehensive sets of materials needed to reproduce curricula faithfully. Our primary research questions underlying this review - based on the participant, intervention, comparison, and outcome (PICO) model [8] - were as follows: (1) what forms of simulation-based curricula designed to teach point-of-care ultrasound to medical learners are currently available, and (2) what evidence exists to show that these are effective in improving learner competency in POCUS techniques?

## Review

Methods

Search Strategy

To address our study questions, we performed a comprehensive search of publications available online through MedEdPORTAL® (https://www.mededportal.org/). Combinatorial search keywords included “simulation,” “curriculum,” “sonography,” “echocardiography,” “bedside ultrasound,” “POCUS,” and “ultrasound education.”

**Figure 1:**
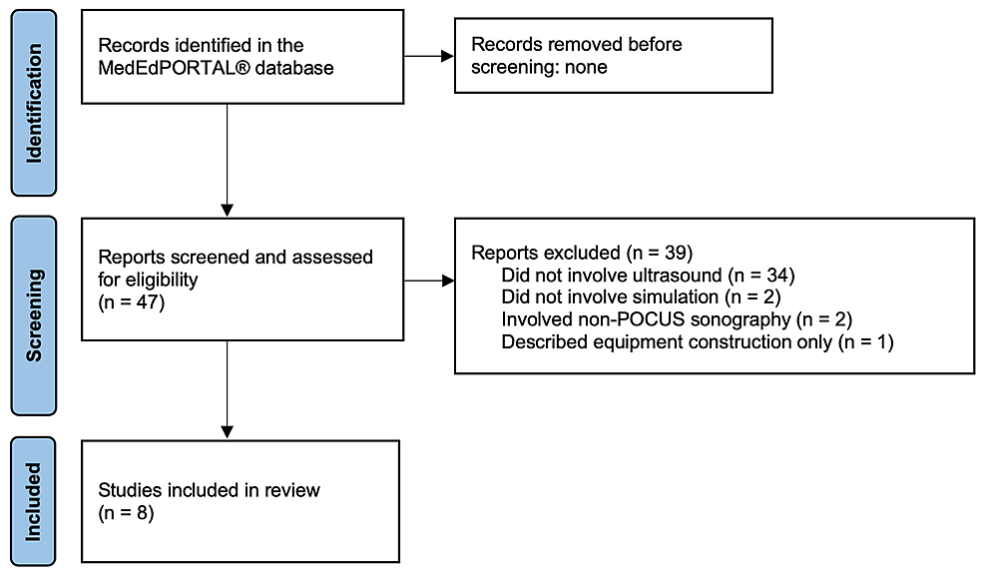
Flow diagram of literature search methodology.

Educational curricula were deemed eligible for inclusion if they were (1) aimed at least in part at improving learners’ POCUS skills and (2) based on one or more simulated cases. Search results were excluded if the identified curricula did not explicitly involve simulation-based instruction, did not include sufficient materials to reproduce the simulated case(s) described, or involved simulations that did not include diagnostic or procedural POCUS techniques. For the purposes of this review, we also excluded applications of ultrasound not traditionally included within POCUS curricula, such as transvaginal ultrasonography, as well as curricula that described only the preparation of ultrasound simulation materials (e.g., low-cost gel simulators) without hands-on POCUS training components. Our complete search methodology is summarized in Figure 1 [9].

Data Collection and Analysis

After target publications were selected, each curricular report and its accompanying materials were parsed by featured pathologies, target audiences, settings, required materials and equipment, and sonographic techniques taught. We also reviewed each publication for reported learner outcomes, particularly for validated and significant improvements in diagnostic or procedural POCUS skill, and for key limitations to the effectiveness or reproduction of each simulation curriculum. All authors of this review have significant experience in point-of-care ultrasound, and the supervisors of this study (EAB and MGC) have successfully completed the Examination of Special Competence in Critical Care Echocardiography (CCEeXAM) by the National Board of Echocardiography (NBE).

Results

Search Results

After reviewing 47 reports returned during an initial search, we identified a total of eight eligible simulation curricula targeted toward teaching POCUS. These curricula are summarized in Table 1, and their characteristics are detailed below.

Underlying Pathologies in Simulation Curricula

The majority (5/8) of the identified simulation curricula centered on the sonographic diagnosis and management of cardiovascular diseases. A diverse curriculum published by Kobayashi et al. created a package of five different ultrasound simulation cases, including right ventricular strain with pulseless electrical activity, pericardial tamponade, hemoperitoneum after trauma, ruptured ectopic pregnancy, and ruptured abdominal aortic aneurysm [10]. In addition to the cases included in this package, we identified three separate ultrasound simulation cases involving cardiac tamponade (Alkhalifah et al., Augenstein et al., and Hitchcock et al.) [11-13] and a more comprehensive focused cardiac ultrasound curriculum that included a diverse array of cardiac pathologies (McConnaughey et al.) [14]. Another case by Sall et al. focused on ultrasound diagnosis of cirrhosis and ascites [15]. A broad medicine-focused curriculum designed for first-year residents published by Vusse et al. targeted a number of ultrasound-guided procedural skills including peripheral venous cannulation, paracentesis, thoracentesis, and lumbar puncture [16]. Finally, a unique obstetric case, developed by Bollag et al., involved a regional anesthesia ultrasound simulation for pain around the time of cesarean delivery in a patient with acute fatty liver of pregnancy [17].

Target Curricular Audiences and Training Environments

All the identified curricula were tailored at least partially toward physician trainees enrolled in graduate medical education programs, and most (6/8) were aimed at acute care audiences. Kobayashi et al. developed their simulation package for emergency medicine trainees within their institution, although the authors stated that the simulations could be more broadly utilized for training undergraduate and graduate medical trainees, staff, nurses, and paramedics. Their simulation materials consequently appear to be most applicable for training clinicians practicing in emergency, intensive care, or perioperative settings [10]. The simulation produced by Alkhalifah et al. was similarly designed for emergency medicine trainees but could be adapted to anesthesiology, critical care, and cardiology trainees [11]. The simulations provided by Hitchcock et al. and Bollag et al. focused primarily on anesthesiology trainees [13,17]. The simulation curricula designed by Sall et al., Vusse et al., and McConnaughey et al. were designed for internal medicine residents on inpatient wards [14-16]. Finally, the tamponade scenario created by Augenstein et al. was uniquely targeted toward pediatric residents [12].

Types of Simulation Equipment Used

The simulation exercises incorporated into the identified curricula commonly contained three elements: a patient model or mannequin, a mock or high-fidelity ultrasound transducer, and a means of rendering or displaying simulated ultrasound images. However, the specific materials used varied considerably. The simulations in the curricular package developed by Kobayashi et al. involve the use of a high-fidelity simulation mannequin with live vital sign monitors coordinated by a technician to reflect dynamic responses to clinical decisions made by the trainee [10]. The curriculum by Sall et al. requires Blue Phantom’s commercially available Paracentesis Ultrasound Training Model® [15], while the curriculum by Bollag et al. requires a generic tissue gel model of an abdomen [17]. Uniquely, the case developed by McConnaughey et al. calls for the use of a mock transducer and computer with a high-fidelity ultrasound simulator validated in prior work [14,18]. The curriculum designed by Vusse et al. involved the creation of a makeshift paracentesis trainer [16]. The curriculum by Alkhalifah et al. was the only one to include a high-fidelity ultrasound simulator in addition to a custom-built pericardiocentesis model [11]. The instructions to build a pericardiocentesis trainer are outlined in a separate paper [19].

Incorporation of Diagnostic Ultrasound Into Simulation Cases

Half (4/8) of the identified simulations aimed to teach diagnostic POCUS interpretation as a skill distinct from procedural ultrasound applications, and only two of these allowed for realistic ultrasound simulation practice. For instance, Alkhalifah et al. constructed a simulation using a high-fidelity simulator that allowed the performance of hands-on bedside ultrasound imaging. However, trainees were not explicitly instructed to perform POCUS to diagnose cardiac tamponade; rather, the exercise allowed learners to discover the utility and importance of performing hands-on bedside ultrasound on their own, as participants that incorporated ultrasound imaging were more successful in managing the standardized patient [11]. The curriculum created by McConnaughey et al. also involved the use of a high-fidelity simulator within a broad cardiac ultrasound module [14]. The remaining curricula did not utilize high-fidelity ultrasound simulators. Instead, video clips of ultrasound images were made available to participants upon request while assessing the patient during the simulation.

Incorporation of Procedural Ultrasound Into Simulations

The use of ultrasound to facilitate the performance of procedures was integrated into four of the identified curricula. Alkhalifah et al. assembled a low-cost pericardiocentesis trainer that allowed dynamic ultrasound-guided procedural practice [11]. During the simulation, the pericardiocentesis trainer was made available when the learner verbalized the need to perform the procedure. The curriculum by Sall et al. utilized a paracentesis trainer for a cirrhotic patient with abdominal pain and ascites, in which the participants performed the procedure under the guidance of faculty who provided real-time feedback and evaluation, and the participants were brought back six months later to demonstrate skill retention on the paracentesis trainer model [15]. The curriculum published by Vusse et al. used multiple dedicated task trainers to teach lumbar puncture, paracentesis, thoracentesis, and peripheral venous cannulation with the aid of POCUS [16]. The curricula by Bollag et al. utilized an oral board-style case in a problem-based learning format, followed by simulated placement of an ultrasound-guided transversus abdominis plane (TAP) block on a gel model trainer, although the procedure was not specifically designed to be performed concurrently with the simulation case.

Additional Materials Required to Conduct Simulations

In addition to the ultrasound-specific equipment detailed previously, the curricular packages found on MedEdPORTAL® also included simulation scripts, patient data, and didactic modules with evaluative components. The curriculum by Kobayashi et al. provides scripts for the simulation role-play of the patient’s complaints and corresponding response to various potential interventions [10]. Their curricular materials also included programming information for the high-fidelity SimMan 3G® by Laerdal Medical so that a technician can readily set up the simulation exercise. Ultrasound videos corresponding to the disease process within each simulated case within the package are provided to be played when the learner requests ultrasound during the simulation. Each of the included cases provides the simulated patient’s presenting history, vital signs, and physiologic responses to the various potential interventions proposed by the learner.

Alkhalifah et al. designed their case for use with a high-fidelity ultrasound simulator, but imaging and ultrasound video footage are also provided in Microsoft PowerPoint form for use in settings without the availability of such simulation equipment [11]. The curricula by Augenstein et al. and Hitchcock et al. both provide scenarios with changes in hemodynamic parameters over time [12,13]. Chest radiographs, electrocardiograms, and bedside ultrasound videos are made available to trainees upon request during the simulation and included for download in the case appendices. The curricula by Augenstein et al. and McConnaughey also include PowerPoint slides summarizing pediatric cardiac tamponade and diverse adult cardiac pathologies in their materials, respectively [12,14].

In addition to a case simulation, the curriculum by Sall et al. provides a pre- and post-simulation quiz, pre-simulation didactic videos, and PowerPoint slides in their case appendices [15]. This is similar to the curriculum by Vusse et al., which includes didactic videos on POCUS basics and ultrasound-guided procedures [16]. The curriculum by Bollag et al. is made available as a large portable document format (PDF) file that includes a didactic component, pre- and post-simulation tests, and a case scenario [17]. The curricula within this review also commonly include a learner competency checklist and post-simulation evaluation form or post-tests.

Reported Learner Outcomes

Ideally, curricula that are made available for learning POCUS will also include evidence of their educational efficacy. While learner satisfaction was generally very high when reported alongside the educational intervention, the majority (7/8) of the identified curricula did not present objective data with respect to learner competency. Only Sall et al. reported that 100% of the learners enrolled achieved a standard of minimum acceptable performance after completing their curriculum, which was determined based on a priori agreement by expert raters. Learners completing this curriculum could repeat the simulated paracentesis as many times as necessary until this standard was reached, and all learners studied were able to meet the standard for successful completion within one hour [15]. The studies by Kobayashi et al. and Bollag et al. did not include outcome data on the efficacy of their curricula [10,17]. The remaining studies included in this review reported highly favorable learner satisfaction scores and/or self-reported confidence ratings after the curricula had been completed, typically employing five-point Likert scale instruments.

Discussion

The use of simulation to teach POCUS is gradually gaining acceptance and momentum, as demonstrated by the variety of open-access curricula published in MedEdPORTAL®. At present, MedEdPORTAL contains only a small number of ultrasound simulation curricula that are directed toward specific patient pathologies, with a preponderance of curricula focusing specifically on cardiovascular pathologies as summarized in Table 1. A multitude of other potential simulations could be designed to teach the use of ultrasonography for diagnosis and evaluation of other organ system diseases including vascular, lung, and abdominal pathologies.

**Table 1:** Summary of published simulation-based ultrasound curricula available on MedEdPORTAL® as of 2021 organized according to publication year and alphabetized by author name.

Study	Pathologies	Intended Audience	Setting	Included Materials	Equipment Needed	Ultrasound Skills	Learner Outcomes	Limitations
Kobayashi et al., 2010 [10]	Right ventricular strain with pulseless electrical activity arrest, pericardial tamponade, hemoperitoneum after trauma, ruptured ectopic pregnancy, ruptured abdominal aortic aneurysm	Undergraduate and graduate medical trainees and staff, nurses, and paramedics; most useful for clinical staff working in emergency medicine, critical care, and anesthesiology departments	Emergency department	Ultrasound pathology video clips, case narrative/role-play scripts and debrief materials, programming for Laerdal Medical’s SimMan®, electrocardiogram and applicable radiographs, evaluation forms for clinical competency and teamwork assessment	SimMan® (Laerdal Medical) mannequin, improvised ultrasound probe	Transthoracic echocardiography, focused assessment with sonography for trauma (FAST) examination	No results or outcomes reported, although competency assessment forms included	No high-fidelity dynamic ultrasound practice included, need to purchase SimMan® simulator, no learner outcomes reported
Bollag et al., 2014 [17]	Peripartum pain, acute fatty liver of pregnancy	Anesthesia residents during an obstetric anesthesia rotation, attending anesthesiologists, certified registered nurse anesthetists, and anesthesia residents	Operating room	Pre-simulation didactic (PDF format), written pre- and post-simulation assessment test, case scenario with integrated instructor oral board-style problem-based learning questions and answers, performance checklist	Tissue gel model of an abdomen, patient volunteer model, ultrasound machine, block needle	Ultrasound-guided transversus abdominis plane (TAP) block	No results or outcomes reported, although performance checklist and learning questions included	Need to build or purchase ultrasound gel model of an abdomen, didactic modules not provided in useful format, case and skills training separated, no learner outcomes reported
Alkhalifah et al., 2016 [11]	Cardiac tamponade	Emergency medicine residents, critical care fellows, cardiology fellows, advanced practice providers in the emergency department or intensive care unit	Emergency department	Ultrasound still and video embedded in a slide presentation, patient case scenario (history, laboratory values, vitals, electrocardiogram, and chest radiograph), instructions and suggestions for faculty/instructor	High-fidelity ultrasound simulator, pericardiocentesis trainer	Transthoracic echocardiography, ultrasound-guided pericardiocentesis	85% of the learners felt that the simulation was effective in teaching diagnosis and management of tamponade; all groups utilizing ultrasound in the diagnostic process were successful	Need to build or purchase pericardiocentesis trainer, simulator programming not provided, no objective assessment of pre- and post-intervention competency
Augenstein et al., 2018 [12]	Pediatric cardiac tamponade	Pediatric emergency medicine fellows, resident physicians in emergency medicine, pediatrics, and family medicine	Pediatric emergency department	Ultrasound still images and videos, case scenario, instructor notes, debrief and evaluation materials, overview of tamponade teaching slides	High-fidelity (non-ultrasound) pediatric simulator mannequin	Pediatric transthoracic echocardiography	High learner satisfaction, high self-reported learner confidence with respect to interpretation of cardiac ultrasound for tamponade (average score of 4.3 out of 5) after simulation	No high-fidelity dynamic ultrasound practice incorporated, simulator programming not provided, no objective assessment of pre- and post-intervention competency
Hitchcock et al., 2018 [13]	Cardiac tamponade, complete heart block during transcatheter aortic valve implantation (TAVI)	Anesthesiology residents, cardiothoracic anesthesia fellows	Operating room	Ultrasound video of tamponade within a slide presentation, electrocardiogram, case narrative, debriefing summary	High-fidelity SimMan® mannequin (non-ultrasound simulator), anesthesia machine	Transesophageal echocardiography	Extremely high self-reported high learner satisfaction, simulation participants later rated qualitatively by faculty as more prepared for and knowledgeable with respect to the TAVI procedure	No high-fidelity dynamic ultrasound practice incorporated, SimMan® programming not provided, need to purchase mannequin, no objective assessment of pre- and post-intervention competency
McConnaughey et al., 2018 [14]	Right ventricular systolic dysfunction or strain, mitral valve prolapse, mitral stenosis, aortic stenosis, bicuspid aortic valve, hypovolemia, pericardial effusion, dilated cardiomyopathy, ischemic cardiomyopathy	Internal medicine residents, nurse practitioners	Medicine wards or intensive care units	Simulator setup instructions, didactic presentation (PowerPoint format), didactic text and outline, pre- and post-tests	High-fidelity mannequin, mock transducer and computer with monitor	Focused cardiac ultrasound (transthoracic echocardiography)	Clear improvements in learner cognitive and psychomotor skill after curriculum, although significance was not directly analyzed within this study	Need to purchase mannequin and equipment, no objective assessment of pre- and post-intervention competency
Sall et al., 2018 [15]	Cirrhosis, ascites, spontaneous bacterial peritonitis	Internal medicine residents, critical care fellows	Any inpatient or outpatient examination room	Paracentesis steps presentation, ultrasound basics presentation, paracentesis instructional video, pre-simulation assessment quiz for ultrasound and paracentesis, case scenario for procedure, two additional post-procedure cases for interpretation and treatment of ascites and SBP	Paracentesis Ultrasound Training Model® (Blue Phantom), ultrasound machine, paracentesis kit	Ultrasound-guided paracentesis	100% of learners achieved a minimum passing standard of competency, as established by national and local experts, within one hour of training; satisfaction was high (average score of 3.88 out of 5)	Need to purchase paracentesis trainer
Vusse et al. 2020 [16]	Pleural effusion, ascites, meningitis, acute hemorrhage	Internal medicine interns during residency orientation	Medicine wards	Didactic videos covering POCUS basics and procedural demonstrations (MP4 format), workshop logistics and rotation schedules, station learning objectives and checklists, pre- and post-simulation questionnaires, setup guide for paracentesis model	Procedure kits for thoracentesis, paracentesis, lumbar puncture, and peripheral venous cannulation; ultrasound machine; prefabricated simulation task trainers; improvised paracentesis model: plastic tray, saline bag, and pigmented silicone pseudoskin	Ultrasound-guided thoracentesis, paracentesis, lumbar puncture, peripheral venous catheter placement	97% of the respondents agreed that simulation was an effective educational tool for procedural training; learners reported satisfaction with hands-on, active, low-pressure learning technique	Need to purchase dedicated task trainers, need to build paracentesis trainer, logistically complex, no objective assessment of pre- and post-intervention competency

Ultrasound-guided procedures may also be practiced using commercial simulators, although few published curricula are available within the MedEdPORTAL® database. For example, Northwestern University’s internal medicine and emergency medicine residency programs utilize Simulab’s CentraLineMan® trainer to teach the insertion of internal jugular and subclavian central lines. Their research suggests that simulation-based teaching can significantly reduce central line complications such as arterial puncture while improving learner confidence [20]. As in other simulation-based ultrasound curricula, however, the lack of easily affordable or accessible high-fidelity equipment remains an obstacle to broader implementation.

Many of the currently existing POCUS-related simulation curricula available in MedEdPORTAL® are more appropriate for learners at the graduate medical education level, at which point some experience with basic ultrasound views has often been acquired. For early medical school students, a workshop format that pairs trainees with experienced instructors and standardized patients may be more appropriate for basic skill acquisition. For example, Blackstock and Carmody published a lecture to teach the basics of ultrasound physics and identify common abdominal structures to medical students [21]. This lecture was then integrated into a standardized patient workshop that ran concurrently with the medical school’s anatomy course and received overwhelmingly positive student feedback. Lim et al. provide a full workshop curriculum addressing the use of POCUS in critical care medicine, which includes didactics for ultrasound basics and vascular access, knobology, thoracic ultrasound, limited echocardiography, and DVT assessment [22]. Although this workshop does not incorporate a simulation component, the addition of a simulation-based training module to such a curriculum may be highly valuable for junior learners. A similar curriculum available in MedEdPORTAL® by Alerhand et al. targets the teaching of renal and bladder ultrasonography in tandem with a preclinical curriculum for first-year medical students, although no true simulation component was included. Learners reported that the use of this curriculum increased their knowledge acquisition with respect to renal anatomy and pathology while also building early comfort with POCUS as a versatile diagnostic modality [23].

Major Drawbacks of Simulation-Based POCUS Curricula

Based on our review, the most identifiable factor limiting the widespread adoption of the identified curricula is the availability of portable, high-fidelity ultrasound simulation systems, which can be prohibitively expensive for many training institutions. Numerous inventive workarounds have been devised, although these alternatives sacrifice a degree of fidelity for convenience and low cost. For example, Damjanovic et al. developed an easy-to-build, low-budget, POCUS simulator that incorporated a radiofrequency identification (RFID) antenna concealed within a mock ultrasound probe [24]. Such an approach using RFID tags, near-field communication (NFC) signals, or quick response (QR) codes could then be attached to existing basic mannequins or standardized patients with images, video clips, or ultrasound loops linked to these tags and displayed on a computer, smartphone, or tablet electronically connected to the mock probe. The significant drawbacks of such low-fidelity ultrasound exercises are (1) that the simulation does not provide the learner with a dynamic understanding of the anatomy and that (2) there is limited opportunity to develop familiarity with practical aspects such as knobology, probe placement, and image acquisition required for effective POCUS implementation. However, many authors specifically acknowledged these limitations in their curricula, emphasizing that simulation-based teaching was intended primarily as an adjunct to bedside instruction involving consenting patients.

As concluded in a prior systematic review, the quality and extent of the data supporting the utility of simulation-based POCUS training remain poor [6]. Within the curricula examined here, only two studies provided objective measures of learner-related performance outcomes. Thus, despite the enthusiasm surrounding POCUS and the use of simulation to build sonographic competency in numerous disciplines, more rigorous and objective studies remain necessary to conclude that simulation-based POCUS training establishes lasting proficiency.

Study Limitations

Although this systematic review uniquely identifies and assesses existing POCUS-related simulation curricula, it is limited to a single educational database. This narrow search strategy was deliberate and intended to maximize the utility of our findings for medical educators, particularly those seeking readily accessible curricular materials that have been utilized in live learning environments previously. Due to limitations in study availability and outcomes reporting, we were unable to directly compare the effectiveness between the identified curricula. It would be informative to examine the comparative effectiveness of POCUS-related simulation curricula in the future when a larger body of curricula becomes available.

## Conclusions

 Point-of-care ultrasound is rapidly emerging as a rapid, accurate, and efficient modality for bedside diagnosis. Simulation-based learning has been demonstrated to improve competency in sonographic image acquisition, interpretation, and integration of this information into medical decision-making. Further rigorous study in the domain of simulation-based ultrasound education remains needed. However, ample opportunities exist for the development and incorporation of more expansive and advanced POCUS simulations into medical school and graduate medical education curricula - particularly as educational supplements to live bedside ultrasound practice - to meet the growing need for structured and standardized ultrasound training.
